# High-Performance One-Dimensional Sub-5 nm Transistors Based on Poly(p-phenylene ethynylene) Molecular Wires

**DOI:** 10.3390/molecules29133207

**Published:** 2024-07-05

**Authors:** Zhilin Chen, Xingyi Tan, Qiang Li, Jing Wan, Gang Xu

**Affiliations:** 1 Department of Physics, Chongqing Three Gorges University, Wanzhou 404100, China; tixonfesc@163.com (Z.C.); wanjing@sanxiau.edu.cn (J.W.); 20040026@sanxiau.edu.cn (G.X.); 2 College of Intelligent Systems Science and Engineering, Hubei Minzu University, Enshi 445000, China; 2012002@hbmzu.edu.cn

**Keywords:** transistor, sub-5 nm gate length, density functional theory, quantum transport simulation

## Abstract

Poly(p-phenylene ethynylene) (PPE) molecular wires are one-dimensional materials with distinctive properties and can be applied in electronic devices. Here, the approach called first-principles quantum transport is utilized to investigate the PPE molecular wire field-effect transistor (FET) efficiency limit through the geometry of the gate-all-around (GAA) instrument. It is observed that the n-type GAA PPE molecular wire FETs with a suitable gate length (*L_g_* = 5 nm) and underlap (*UL* = 1, 2, 3 nm) can gratify the on-state current (*I_on_*), power dissipation (*PDP*), and delay period (*τ*) concerning the conditions in 2028 to achieve the higher performance (HP) request of the International Roadmap for Device and Systems (IRDS, 2022 version). In contrast, the p-type GAA PPE molecular wire FETs with *L_g_* = 5, 3 nm, and *UL* of 1, 2, 3 nm could gratify the *I_on_*, *PDP*, and *τ* concerning the 2028 needs to achieve the HP request of the IRDS in 2022, while *L_g_* = 5 and *UL* = 3 nm could meet the *I_on_* and *τ* concerning the 2028 needs to achieve the LP request of the IRDS in 2022. More importantly, this is the first one-dimensional carbon-based ambipolar FET. Therefore, the GAA PPE molecular wire FETs could be a latent choice to downscale Moore’s law to 3 nm.

## 1. Introduction

A field-effect transistor (FET) is recognized as one of the most important inventions in the last century [1]. Now, it is going in time with one-dimensional and two-dimensional semiconductor materials [2,3,4,5]. Due to their excellent electrical properties, carbon-based field-effect transistors (FETs) have been studied in depth in the last decade [6,7,8,9,10]. Carbon nanotube transistors were the first obtained carbon-based FETs fabricated by Tans et al. [6] using metal single-wall carbon nanotubes. Soon after, they constructed a FET comprising a semiconducting single-wall carbon nanotube linked with two metal electrodes [7]. The carbon nanotube was changed from a conducting to an insulating phase after exerting a voltage on the gate electrode. Multi-wall carbon nanotube-based FETs were also obtained by Martel et al. [11], while the transport was dominated by holes and appeared diffusive. In the last decade, back-gated or new top-gated instrument structures were adopted to construct sub-10 nm gate-length (*L_g_*) carbon nanotube FETs [12,13]. Recently, Xu et al. [14] theoretically discovered that gate-all-around (GAA) carbon nanotube FETs could fulfill the International Roadmap for Semiconductors (ITRS) 2028 HP goal in the *L_g_* = 2 nm node regarding the on-state current (*I_on_*), delay time (*τ*), and power consumption (*PDP*). Appenzeller et al. [15]. also presented band-to-band tunneling in carbon nanotube FETs, except for the conventional metal oxide semiconductor FET. Although tremendous progress has been achieved in carbon nanotubes for FET devices, the main issue is the restricted control of the chirality and diameter of nanotubes (and the related electronic bandgap) [10]. Furthermore, carbon nanotubes have a small band gap (E_g_ < 0.9 eV); hence, they are not appropriate for LP applications [9]. The other type of carbon-based FET is graphene FET, which was first reported in 2007 and represented a significant milestone, and progress has been rapid since then [16]. Meric et al. [17]. first observed the saturating transistor features in a graphene FET. Despite the significant development in graphene for FET devices, it cannot be employed as a semiconductor material due to the lack of a bandgap in graphene [18,19,20]. One of the most optimal approaches to opening up a bandgap in graphene is the constructional restriction into graphene nanoribbons (GNRs) [18]. Wang et al. [21] verified sub-10 nm-wide GNR FETs and attained an *I_on_*/*I_off_* rate of up to 10^6^ and an I_on_ density of as high as 2000 μA/μm. In 2013, Bokor and colleagues [22] first presented the seven-armchair GNR (AGNR) transfer provided under ultrahigh vacuum to SiO_2_/Si substrates to fabricate short-channel FETs. Due to the GNRs’ small width and significant bandgap, the Schottky barriers at the electrodes significantly dominated the electronic transport. Low band-gap 9- and 13-AGNRs in FET instruments with a short channel were employed to alleviate the Schottky barriers and enhance the efficiency of the instrument [23]. Transferring chemical vapor deposition-synthesized 5-, 7- and 9-AGNRs concerning FET devices was presented by Sakaguchi et al. [24]. However, the instruments exhibited an ambipolar transistor nature containing carrier mobilities from 10^−6^ through 10^−4^ cm^2^ V^−1^ s^−1^ and an *I_on_*/*I_off_* rate < 5 [18]. Therefore, neither carbon nanotube transistors nor GNR FETs are suitable for actual applications. So, finding new carbon-based FET devices is still a topic of interest. Fortunately, Tsumura et al. [25]. conducted a pioneering study in which they successfully manufactured a FET using polythiophene molecular wire as the semiconductor material. Their work opens a door for the fabrication of FETs by using molecular wire. Recently, Shu et al. [26]. fabricated single arrays of PPE molecular wires with a bandgap of 1.85 eV, providing a larger bandgap than carbon nanotubes. Thomas et al. [27] calculated the electric structure of PPE molecular wires with a small effective mass (0.11 m_0_ for electrons, 0.12 m_0_ for holes), meaning conceivably larger carrier mobilities than GNRs. A more proper bandgap and a small effective mass may overcome the insufficiency of the carbon nanotubes and GNRs. Therefore, it is meaningful to investigate PPE molecular wire FETs. The current work constructed GAA FETs with a sub-5 nm channel length via PPE molecular wire and employed the ab initio quantum transport technique to verify their theoretical efficiency. The outcomes demonstrated that p-type PPE molecular wire FETs with a suitable underlap (*UL*) could fulfill the *I_on_*, *τ*, and *PDP* needs in 2028 when the International Roadmap for Device and Systems (IRDS, 2022 version) [21] concerns the HP usages, with *L_g_* of 5 and 3 nm. At the same time, the n-type GAA PPE molecular wire FETs with a suitable *L_g_* = 5 nm and *UL* = 1, 2, 3 nm could gratify the *I_on_*, *PDP*, and *τ* concerning the 2028 needs to achieve the HP request of the IRDS in 2022. Accordingly, PPE molecular wire FETs can scale down the same law to 3 nm.

## 2. Results and Discussion

**Figure 1:**
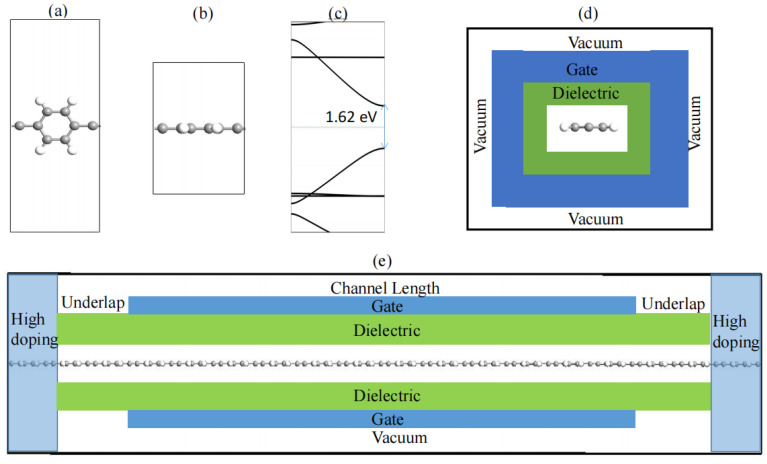
Schematic structure description of the PPE molecular wires: (**a**) top perspective and (**b**) side perspective. (**c**) The PPE molecular wires’ band structures. Schematic description of the GAA PPE molecular wire FETs: (**d**) side perspective and (**e**) cutaway perspective.

The crystal structures of the PPE molecular wires are plotted in Figure 1, showing (a) the top perspective and (b) the side perspective. The calculated lattice parameter of the PPE molecular wire was 6.92 Å, compatible with the experimental scanning tunneling microscopy result of 6.7 ± 0.2 Å by Shu et al. [26]. The calculated lattice parameter of the PPE molecular wire was slightly bigger than the theoretical calculating value of 6.88 Å by Shu et al. [26]. Figure 1c depicts that the PPE molecular wire was a direct bandgap semiconductor of 1.62 eV, presented with the scanning tunneling spectroscopy measurement outcomes provided by Shu et al. [26] (with a 1.60 eV bandgap). The bandgap of the PPE molecular wire was slightly smaller than the theoretical calculating value of 1.85 eV by Shu et al. [26]. In terms of the equation $\frac{1}{m^{*}}=\frac{1}{\hbar }\frac{d^{2}E}{dk^{2}}$, the electron effective mass (m_e_*) of the PPE molecular wires was 0.105 *m*_0_ along the Γ-Z direction, while the hole effective mass (m_h_*) value was 0.114 *m*_0_ along the Γ-Z direction. These calculated values are in good agreement with what Thomas et al. [27] reported. Figure 1d,e describe the sub-5 nm *L_g_* GAA PPE molecular wires constructed by inherent PPE molecular wires (heavily n-doped or p-doped) as channels (electrodes). A supply voltage (*V_dd_*) was chosen as 0.64 V, and an equivalent oxide thickness form was selected as 0.41 nm. A charge recovery mechanism providing additional charges was employed to dope the electrodes. Table S1 (in the Supplementary Materials) shows the calculated maximum current of the GAA PPE molecular wire FETs (*L_g_* = 5 nm, *UL* = 3 nm). Since on-state higher currents are available in the IRDS in 2022 and better gate control was obtained, the doping immersions were selected as 1 × 10^7^ m^−1^.

**Figure 2:**
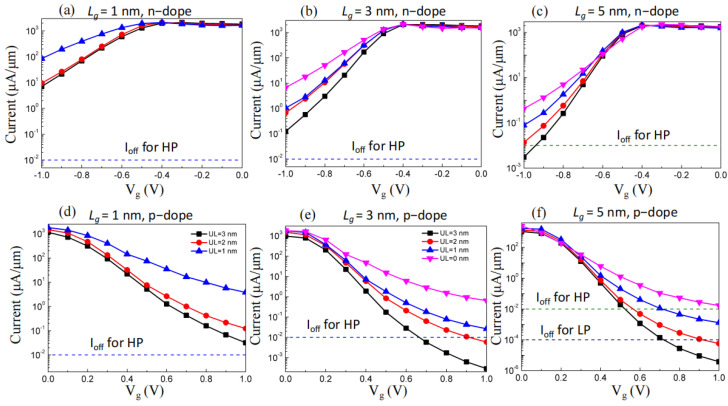
The attributes related to the current voltage of gate-all-around FETs. (**a**) N-doped, *L_g_* = 1 nm; (**b**) n-doped, *L_g_* = 3 nm; (**c**) n-doped, *L_g_* = 5 nm; (**d**) p-doped, *L_g_* = 1 nm; (**e**) p-doped, *L_g_* = 3 nm; (**f**) p-doped, *L_g_* = 5 nm. The value of 0.64 V is biased voltage. The square, circle, up triangle, and down triangle represent *UL* = 0, 1, 2, and 3 nm, respectively.

Figure 2 presents the sub-5 nm *L_g_* GAA PPE molecular wire FETs’ transfer characteristics (perimeter-normalized). The plots show that all the currents decreased while the absolute value of voltage increased, i.e., when the voltages were small, the currents slowly decreased as the voltages increased; when the voltages reached a specific value, the currents decreased sharply as the voltages increased; and the currents slowly decreased as the voltages increased in the end, thus indicating a typical diode effect. To describe this effect in depth, the subthreshold swing (*SS*) of the GAA PPE molecular wire FETs was calculated as a critical factor that determines the gate control capability, defined by [1,28,29]:

*SS* = ∂*V*_g_/∂(lg*I*_ds_)

**Figure 3:**
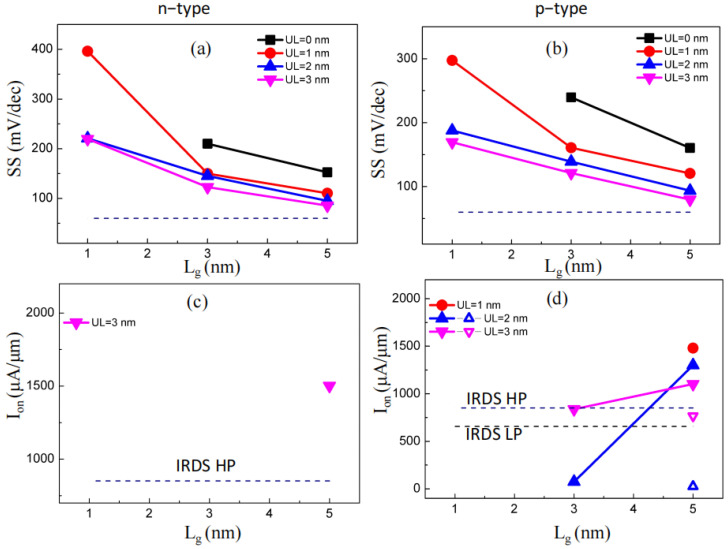
(**a**,**b**) *SS* concerning the *L_g_* with various underlap lengths. (**c**,**d**) On-state current (*I_on_*) of the PPE molecular wire FETs versus the gate length for PPE molecular wire FETs with various values of *L_g_*. The dashed lines indicate the adjusted values of 851 and 656 μA/μm assigned to the HP and LP on-state currents, respectively, based on the benchmarks. The solid symbol represents the HP situation, while the open symbol represents the LP situation.

**Figure 4:**
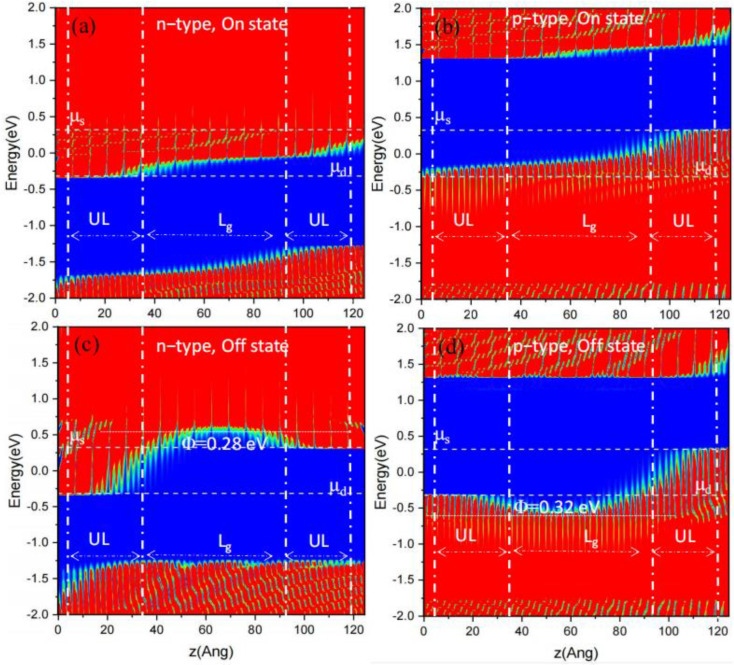
The LDOS concerning L_g_ assigned to 5 nm and UL = 3 nm at on- and off-state. (**a**) N-type, on-state; (**b**) p-type, on-state; (**c**) n-type, off-state; (**d**) p-type, off state. μ_s_ and μ_d_ describe the electrochemical potentials of the source and drain.

When the subthreshold region is a concern, a higher *SS* reflects the weaker gate control capability. Figure 3a,b depict that a subthreshold swing of the GAA PPE molecular wire FETs generally decreased when growing *L_g_* and *UL*. *SS* grew when scaling down *L_g_* from 5 and 1 nm with *UL* = 3 nm, namely, from 86/80 to 220/169 mV/dec for n-type/p-type PPE molecular wire FETs. *SS* decreased while increasing the *UL* at the same gate length. For instance, *SS* changed from 153/160 to 86/80 mV/dec for n-type/p-type PPE molecular wire FETs for *UL* scaling up from 0 to 3 nm (*L_g_* = 5 nm). In a word, *SS* of GAA PPE molecular wire FETs generally dropped when growing the *L_g_* and *UL*. To further explain the gate control capability of the devices, the *L*_g_ = 5 nm with *UL* = 3 nm GAA PPE molecular wire FETs were used for the HP usages. Figure 4 depicts the resolved position of a local density of states (LDOSs) at on- and off-states. The energy activating the electron *Φ_B_* describes the difference in the energy between the maximum bases of the conduction band and the Fermi level. The *Φ_B_* increased from 0 eV to 0.28 and 0.32 eV for n-type and p-type devices, respectively, assisting in obtaining the off-state.

The on-state current describes the current transfer properties at the on-state gate voltage (*V_g,on_*), a critical parameter concerning FETs. *V_g,on_* and *V_g,off_* describe the on-state and off-state gate voltages, respectively. *V_dd_* = *V_b_* (bias voltage) was assumed [30]. The off-state current (*I_off_*) was assigned to 0.01 µA/µm (according to the HP needs) and 1 × 10^−4^ µA/µm (according to the LP needs) based on the IRDS. As presented in Figure 2, only the p-type PPE molecular wire FETs (*L*_g_ = 5 nm, *UL* = 3, 2 nm) could reach the LP off-state. However, only the *I_on_* (equal to 889.5 µA/µm) of *L*_g_ = 5 nm and *UL* = 3 nm was larger than LP (656 µA/µm). As presented in Figure 2c and Figure 3c, only *L_g_* = 5 nm and *UL* = 3 nm devices could meet the on-state standards concerning the IRDS’ HP needs (851 µA/µm) for n-type PPE molecular wire FETs. Similarly, for p-type PPE molecular wire FETs, the case of a *L_g_* = 5 nm, *UL* = 1, 2, 3 nm; *L_g_* = 3 nm, *UL* = 2, 3 nm device could meet the on-state standards concerning the IRDS’ HP needs, as shown in Figure 2d–f and Figure 3d. In summary, p-type PPE molecular wire FETs are appropriate for HP devices and could scale Moore’s law below 3 nm.

The effective delay time *τ* specifies the GAA PPE molecular wire FETs’ switching rate, which can be obtained as follows.

**Figure 5:**
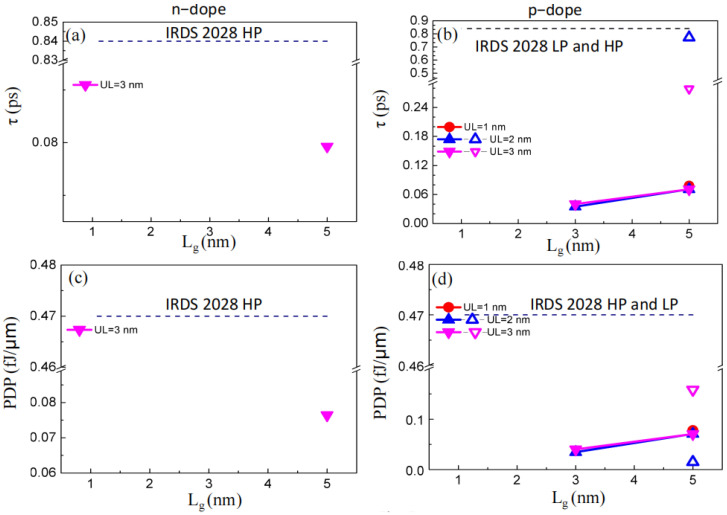
(**a**,**b**) Inherent delay time versus the gate length for PPE molecular wire FETs with various *L_g_* scores. The purposes of HP/LP = 0.84 ps, (**c**,**d**) PDP versus the gate length for PPE molecular wire FETs with various L_g_ values are described by the dashed lines, which present HP/LP = 0.47 fJ/μm.

*τ* = *C_t_V_dd_*/*I_on_*,

where *C_t_* describes the total capacitance, where *C_t_* = *C_g_* + *C_f_*, *C_f_*, and *C_g_* indicate the fringing and gate capacitances, respectively; *C_g_* = ∂*Q_ch_*/∂*V_g_* and *C_f_* become two times the *C_g_*; and *Q_ch_* describes the fully charged main region. Figure 5a,b denote the obtained scores for *τ*. The *τ*’s obtained values (0.03–0.23 ps) concerning 3 and 5 nm *L_g_* GAA PPE molecular wire FETs could fulfill the needs of the 2028 HP (0.84 ps concerning IRDS) about HP instruments. The delay time of the simulated sub-5 nm *L_g_* GAA PPE molecular wire FET was below the theoretically set bound of the binary logic switch controlled with a barrier (0.04 ps) [31,32] since the Shannon–von Neumann–Landauer description was invalid for instruments with *L_g_* < 5 nm, where the process of tunneling cannot be ignored. The probability of the spontaneous transition through the channel barrier *P_erro_* can be described as follows [33]:

$$P_{erro}=\exp(-\frac{E_{b}}{kT})+\exp(-\frac{2L_{b}\sqrt{2m*E_{b}}}{\hbar })-\exp(-\frac{\hbar E_{b}+2L_{b}kT\sqrt{2m*E_{b}}}{\hbar kT})$$

where *E_b_* describes the height of the energy barrier, *k* indicates a constant called Boltzmann, *T* describes the temperature, *L_b_* indicates the width of the barrier, m* describes the efficient mass, and *ℏ* indicates the lowered Planck constant. To ensure detectable phases, *P_erro_* should be higher than 0.5 in the off-state; that is, the minimum of *E_b_* can be estimated as $kT\ln2+\frac{\hbar^{2}{\left(\ln2\right)}^{2}}{8m*L_{b}^{2}}$, which can exceed the limit of Shannon–von Neumann–Landauer (0.017 eV). Now, the shortest switching duration is approximated as *ℏ*/*E_b_*, below 0.04 ps. For instance, in the presented 5 nm *L_g_* GAA PPE molecular wire FET, *E_b_* was 0.28/0.32 eV in the off-state of n-type/p-type PPE molecular wire FET, as presented in the LDOS of Figure 4, and the delay time limit was 0.0024/0.0021 ps. The obtained values of *C*_t_ are presented in Table S2. The *C_t_* values of GAA PPE molecular wire FETs (*L_g_* = 3, 5 nm) were smaller than the HP = 0.6 fF/μm goal. In summary, the effective delay time and the total capacitance of p-type PPE molecular wire FETs (*L_g_* = 3, 5 nm for p-type and *L_g_* = 5 nm for n-type) are appropriate for HP devices.

*PDP*, the next critical factor determining the FETs’ switching energy, is described as *PDP* = *V_dd_I_on_τ*. It determines the consumed energy per switching event. Figure 5c,d and Table S2 indicate that the obtained *PDP* (0.008−0.177 fJ/μm) of the sub-5 nm *L_g_* PPE molecular wire FETs was below the 2028 HP needs (0.47 fJ/μm for IRDS). Moreover, the *PDP* of the PPE molecular wire FETs decreased while scaling *L_g_* and increasing *UL*. In summary, the *PDP* of PPE molecular wire FETs (*L_g_* = 3, 5 nm for p-type and *L_g_* = 5 nm for n-type with appropriate *UL*) are appropriate for HP devices.

**Figure 6:**
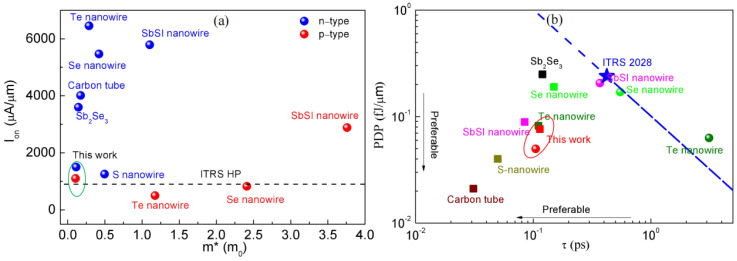
(**a**) The FETs’ on-state current concerning HP usage, with *L_g_* = 5 nm versus the m* of PPE molecular wire FETs and various one-dimensional channel materials. Ab initio quantum transport simulations are employed to acquire all the data. (**b**) The PDP versus τ of the n-type (square points) and p-type (sphere points) of the 5 nm *L_g_* FETs using various channel materials with one-dimensional structures concerning the HP usages. The standard is against the IRDS for the 2028 field of view. PDP = EDP/τ is described by dashed lines.

To determine the critical parameters influencing *I_on_*, Figure 6a shows the mutual relationship between the efficient mass (m*) and *I_on_* of previous studies on one-dimension semiconductor FETs with 5 nm *L_g_*, involving the Te nanowire [34], Se nanowire [35], carbon nanotube [14], Sb_2_Se_3_ nanowire [36], S nanowire [32], and SbSI nanowire [33]. As we know, in Figure 6a, *I_on_* = *neυ*, where *n* and *υ* describe the carrier concentration and the velocity, respectively. Therefore, *I_on_* is primarily related to *υ* and *n*. Now, *υ* is defined as $\upsilon =\frac{\hbar k}{m^{*}}$, where ℏ describes the lowered Plank constant, and *k* describes the wave vector. Hence, *υ* changes inversely with m*. Besides, *n* denotes the ratio of the density of states (DOS), described by $DOS=\frac{g_{s}g_{u}}{2\pi \hbar^{2}}\sqrt{m_{x}^{2}m_{y}^{2}}$, where *g_s_* and *g_v_* indicate the degeneracies related to spin and valley, and *m*_x_ and *m*_y_ describe the efficient masses related to transverse and transport, respectively. Concerning the HP case in Figure 6, *υ* is a critical parameter for *I_on_* for small values of m*. The *I_on_* of PPE molecular wire FETs is slightly bigger than the IRDS standard because the electrons are localized in a -C≡C- bond [26]. PPE molecular wire FETs are the only one-dimensional devices that can work as p-type. *EDP* describes the energy efficacy of the equipment and represents the relationship between *τ* and *PDP* via *EDP* = *τ* × *PDP*. Figure 6b presents one-dimensional semiconductor FETs with 5 nm *L_g_* with EPD, involving Te nanowire [29], Se nanowire [30], carbon nanotubes [9], Sb_2_Se_3_ nanowire [31], S nanowire [32], and SbSI nanowire [33]. The dot at the bottom left indicates the devices with a lower *EDP*. The perspective concerning the IRDS 2028 can be utilized as a benchmark. The PPE molecular wire FETs with 5 nm *L_g_* considerably satisfy the IRDS’s purposes concerning HP usage. More interestingly, the PPE molecular wire FETs outperformed the Te nanowire, Se nanowire, and Sb_2_Se_3_ nanowire FETs, and outperformed the carbon tube and S nanowire FETs.

## 3. The Approach and the Model

Software called the Atomistix ToolKit 2019 [37] was utilized to verify the sub-5 nm *L_g_* PPE molecular wire FETs’ transport characteristics. The drain current could be attained using an equation called Landauer–Bűttiker, presented by Equation (1) [38,39]:

(1) $$I\left(V_{b},V_{G}\right)=\frac{2e}{h}\int_{-\infty }^{+\infty }\left\{T\left(E,V_{b},V_{G}\right)\left[f_{S}\left(E-\mu_{S}\right)-f_{D}\left(E-\mu_{D}\right)\right]\right\}dE$$

where the gate voltage and its bias are described by *V_G_* and *V_b_*, respectively. *T*(*E*, *V_b_*, *V_G_*) describes the coefficient average of *k*-dependent transmissions in the Brillouin area *f_S_*_(*D*)_ and *μ_S_*_(*D*)_ describe the source, drain, and Fermi–Dirac distribution mappings of the electrochemical potential (EP). $T_{k_{//}}\left(E\right)$ can be described as the following [40]:

(2) $$T_{k_{//}}\left(E\right)=\mathrm{Tr}[\Gamma_{k_{//}}^{l}(E)G_{k_{//}}(E){\;\Gamma }_{k_{//}}^{r}(E)G_{k_{//}}^{†}(E)]$$

where *k*_//_ describes a point of a reciprocal lattice vector vertical to the transmission orientation (across a direction parallel to a surface). $G_{k_{//}}(E)$ and $G_{k_{//}}^{†}(E)$ indicate the green functions (GFs) in retarded and advanced forms, respectively. The GF can be written as the following:

(3) $${G_{k}}_{//}(E)=[(E+i\eta^{+})I-{H_{k}}_{//}-\Sigma_{k//}^{S}-\Sigma_{k//}^{D}{]}^{-1}$$

where *I*, *H_k//_*, *E*, and *η*^+^ describe the identity and Hamiltonian channel matrices, the energy, and a small nonnegative coefficient, respectively. $\Sigma_{k//}^{S(D)}$ describes the self-energy, reflecting the link between source, drain, and channel. $\Gamma_{k_{//}}^{l(r)}\left(E\right)=i(\sum_{k_{//}}^{l(r)}\;-{\left(\sum_{k_{//}}^{l(r)}\right)}^{†})$ describes the level widening induced by the left or right source or drain electrodes in the self-energy form $\sum_{k_{//}}^{l(r)}$. Generalized Gradient Approximation (GGA) is described by Perdew–Burke–Ernzerhof mapping [41], reflecting the interconnection between exchange and correlation. GGA-based DFT with a single-electron estimation represents the electronic framework due to the strict monitoring of electron–electron interaction caused by doping carriers [42,43]. The Brillouin area utilizes a 1 × 1 × 100 Monkhorst Pack. Thus, the electron temperature and the cutoff energy of real space were assigned 300 K and 100 Hartree. The transverse, vertical, and transport directions’ boundary requirements were chosen based on Newman, Newman, and Dirichlet kinds, respectively.

## 4. Conclusions

The current paper verified the performance of sub-5 nm *L_g_* GAA PPE molecular wire FETs via the *quantum ATK* version P-2019.03 atomic-scale modeling software, combining the density functional theory (DFT) with the nonequilibrium Green’s function. The theoretical simulations demonstrated that the n-type GAA PPE molecular wires FETs (*L_g_* = 5 nm, *UL* = 3 nm) and the p-type GAA PPE molecular wires FETs (*L_g_* = 5 nm, *UL* = 1, 2, 3 nm; *L_g_* = 3 nm, *UL* = 3 nm) could gratify the *I_on_*, *PDP*, and *τ* for the 2028 needs for the HP request of the 2022 IRDS. At the same time, the p-type GAA PPE molecular wire FETs (*L_g_* = 5 nm, *UL* = 3 nm) could fulfill the *I_on_*, *PDP*, and *τ* for the 2028 needs for the LP request of the 2022 IRDS. More importantly, this is the first report about one-dimensional carbon-based ambipolar FETs. Our theoretical work puts forward a new kind of one-dimensional materials, i.e., PPE molecular wires, which can be utilized as potential material to reach a Moore’s Law of less than 3 nm.

## Data Availability

The data presented in this study are available in article and Supplementary Materials.

## Supplementary Materials

The following supporting information can be downloaded at: https://www.mdpi.com/article/10.3390/molecules29133207/s1, Table S1. The doping concentration and the maximum currents. Table S2. Criteria of the sub-5 nm L_g_ GAA PPE molecular wires FETs’ ballistic efficiency versus the 2028 requirements of the IRDS 2020 for the HP applications.
