# Supplementary material for: High-Performance One-Dimensional Sub-5 nm Transistors Based on Poly(p-phenylene ethynylene) Molecular Wires
Source: Molecules. 2024 Jul 5;29(13):3207. doi: 10.3390/molecules29133207 (PMC11243332; doi:10.3390/molecules29133207)
Supplement: Supplementary file 1 [file molecules-29-03207-s001.zip › molecules-3013083-supplementary.pdf]

# High-Performance One-Dimensional Sub-5 nm Transistors Based on Poly(p-phenylene ethynylene) Molecular Wires

**Zhilin Chen [1], Xingyi Tan [1,*], Qiang Li [2], Jing Wan [1] and Gang Xu [1]**

[1] Department of Physics, Chongqing Three Gorges University, Wanzhou 404100, China; tixonfesc@163.com (Z.C.); wanjing@sanxiau.edu.cn (J.W.); 20040026@sanxiau.edu.cn (G.X.)
[2] College of Intelligent Systems Science and Engineering, Hubei Minzu University, Enshi 445000, China; 2012002@hbmzu.edu.cn
* Correspondence: tanxy@sanxiau.edu.cn

**Table S1.** The doping concentration and the maximum currents

| Doping type | Doping concentration (/m) | Maximum currents (μA/μm) |
|---|---|---|
| P-type | $1\times10^{6}$ | 60.0 |
| | $1\times10^{7}$ | 1102.6 |
| | $1\times10^{8}$ | 12591.5 |
| N-type | $1\times10^{6}$ | 112.1 |
| | $1\times10^{7}$ | 1502.0 |
| | $1\times10^{8}$ | 16984.2 |

**Table S2.** Criteria of the sub-5 nm $L_g$ GAA PPE molecular wires FETs' ballistic efficiency versus the 2028 requirements of the IRDS 2020 for the HP applications.

| | $L_g$ (nm) | UL (nm) | *SS* (mV/dec) | $I_{off}$ (μA/μm) | $I_{on}$ (μA/μm) | $I_{on}/I_{off}$ | $C_t$ (fF/μm) | $\tau$ (ps) | PDP (fJ/μm) |
|---|---|---|---|---|---|---|---|---|---|
| **N-type** | 5 | 0 | 153 | 0.01 | - | - | - | - | - |
| | | 1 | 110 | 0.01 | 1966.15 | $1.97\times10^5$ | 0.096 | 0.094 | 0.118 |
| | | 2 | 95 | 0.01 | 1904.16 | $1.90\times10^5$ | 0.079 | 0.080 | 0.097 |
| | | 3 | 86 | 0.01 | 1502.03 | $1.50\times10^5$ | 0.062 | 0.080 | 0.076 |
| | 3 | 0 | 210 | 0.01 | - | - | - | - | - |
| | | 1 | 150 | 0.01 | - | - | - | - | - |
| | | 2 | 145 | 0.01 | - | - | - | - | - |
| | | 3 | 123 | 0.01 | - | - | - | - | - |
| | 1 | 1 | 396 | 0.01 | - | - | - | - | - |
| | | 2 | 221 | 0.01 | - | - | - | - | - |
| | | 3 | 220 | 0.01 | - | - | - | - | - |
| **P-type** | 5 | 0 | 160 | 0.01 | 1202.91 | $1.20\times10^5$ | 0.144 | 0.230 | 0.177 |
| | | 1 | 121 | 0.01 | 1725.01 | $1.73\times10^5$ | 0.070 | 0.078 | 0.086 |
| | | 2 | 94 | 0.01 | 1300.23 | $1.30\times10^5$ | 0.048 | 0.071 | 0.059 |
| | | 3 | 80 | 0.01 | 1102.62 | $1.10\times10^5$ | 0.041 | 0.071 | 0.050 |
| | 3 | 0 | 240 | 0.01 | - | - | - | - | - |
| | | 1 | 161 | 0.01 | 975.7 | $9.76\times10^4$ | 0.036 | 0.068 | 0.043 |
| | | 2 | 139 | 0.01 | 1420.9 | $1.42\times10^5$ | 0.026 | 0.035 | 0.032 |
| | | 3 | 121 | 0.01 | 967.6 | $9.68\times10^4$ | 0.020 | 0.040 | 0.025 |
| | 1 | 1 | 297 | 0.01 | - | - | - | - | - |
| | | 2 | 188 | 0.01 | - | - | - | - | - |
| | | 3 | 169 | 0.01 | 262.6 | $2.63\times10^4$ | 0.007 | 0.053 | 0.009 |
| **IRDS HP 2028** | 12 | - | - | 0.01 | 851 | $8.51\times10^4$ | 0.37 | 0.84 | 0.47 |

$L_g$: the gate length. UL: the underlap length. *SS*: the subthreshold swing. $I_{off}$: the off-state current. $I_{on}$: the on-state current. $C_g$: the gate capacitance. τ: the delay time. PDP: the power dissipation.
